# Evaluation of the use of untargeted metabolomics in the safety assessment of genetically modified crops

**DOI:** 10.1007/s11306-020-01733-8

**Published:** 2020-10-09

**Authors:** Mohamed Bedair, Kevin C. Glenn

**Affiliations:** Bayer Crop Science, Saint Louis, MO USA

**Keywords:** Crop safety assessment, Genetically modified crops, Untargeted metabolomics

## Abstract

**Background:**

The safety assessment of foods and feeds from genetically modified (GM) crops includes the comparison of key characteristics, such as crop composition, agronomic phenotype and observations from animal feeding studies compared to conventional counterpart varieties that have a history of safe consumption, often including a near isogenic variety. The comparative compositional analysis of GM crops has been based on targeted, validated, quantitative analytical methods for the key food and feed nutrients and antinutrients for each crop, as identified by Organization of Economic Co-operation and Development (OCED). As technologies for untargeted metabolomic methods have evolved, proposals have emerged for their use to complement or replace targeted compositional analytical methods in regulatory risk assessments of GM crops to increase the number of analyzed metabolites.

**Aim of Review:**

The technical opportunities, challenges and strategies of including untargeted metabolomics analysis in the comparative safety assessment of GM crops are reviewed. The results from metabolomics studies of GM and conventional crops published over the last eight years provide context to enable the discussion of whether metabolomics can materially improve the risk assessment of food and feed from GM crops beyond that possible by the Codex-defined practices used worldwide for more than 25 years.

**Key Scientific Concepts of Review:**

Published studies to date show that environmental and genetic factors affect plant metabolomics profiles. In contrast, the plant biotechnology process used to make GM crops has little, if any consequence, unless the inserted GM trait is intended to alter food or feed composition. The nutritional value and safety of food and feed from GM crops is well informed by the quantitative, validated compositional methods for list of key analytes defined by crop-specific OECD consensus documents. Untargeted metabolic profiling has yet to provide data that better informs the safety assessment of GM crops than the already rigorous Codex-defined quantitative comparative assessment. Furthermore, technical challenges limit the implementation of untargeted metabolomics for regulatory purposes: no single extraction method or analytical technique captures the complete plant metabolome; a large percentage of metabolites features are unknown, requiring additional research to understand if differences for such unknowns affect food/feed safety; and standardized methods are needed to provide reproducible data over time and laboratories.

## Introduction

The 1975 Asilomar Conference on Recombinant DNA initiated a public discussion of the potential benefits and hazards for the emerging field of molecular biotechnology (Berg et al. 1975). Building from the Asilomar conference, in 1992 the US FDA conjectured, in the absence of direct experience or data, that the process of making GM crops might induce inadvertent mutations that could, in turn, activate dormant metabolic pathways (Kessler et al., 1992). Further speculating whether GM plants might gain an ability to make toxins or toxic intermediates as an unintended consequence of recombinant DNA technology being applied to crop plants. In 1993, the OECD developed a basis for assessing GM crops by stating that there should be “…*a reasonable certainty that no harm will result from intended uses under the anticipated conditions of consumption*…”(OECD, 1993). In support of this principle, the OECD developed a series of crop-specific guidance documents defining key analytes to be assessed for each crop, based on food/feed safety and/or nutritional perspectives. Subsequently, a number of guidance documents and regulations were developed for GM crops to evaluate both their safety for the environment and for food/feed safety for human and animal consumption (Codex Alimentarius 2009; EFSA 2006).

Since the first commercialization of GM crops in 1995, the rapid adoption rates of GM crops reflect the value farmers have derived from this technology (ISAAA, 2017). The commercial production of GM crops uses multiple inter-woven steps throughout the process to develop new varieties that help ensure safety (Glenn et al. 2017; Herman et al. 2019). Therefore, it is noteworthy that 44 years after the Asilomar conference attendees first debated the risks and benefits of biotechnology, over 6000 peer-reviewed publications have not documented examples of unsafe effects of commercialized GM crops. Globally from 1992 to 2017, 40 countries (including the EU 28, counted as one) have granted 1,995 food approvals 1,338 feed approvals, and 800 cultivation approvals for 498 events of 29 crops (ISAAA, 2017). In each approval, the conclusion has been that, outside of the intended change from the GM trait(s), the GM crop is “as safe as” the conventional varieties for that crop species, with no findings of adverse unintended changes resulting from the use of biotechnology to improve plants. Importantly, a recent meta-analysis of publications over the past 21 years with GM maize documented increased yields accompanied by reductions in dangerous food contamination, such as mycotoxins, fumonisin, and trichothecenes. (Pellegrino et al. 2018).

To assess the safety and nutritional value of foods and feeds from GM crops, a comparative process is followed in which the composition of the GM crop is compared to a near isogenic conventional counterpart (Codex Alimentarius 2009; Paoletti et al. 2008; Prado et al. 2014; Privalle et al. 2012). The specific compositional components assessed in these comparative studies are defined by crop-specific OECD consensus documents that identify the critical food and feed nutritional and anti-nutritional components that need to be quantified. The OECD recommended components include crop macronutrients and micronutrients and comprise greater than 95% dry matter of the crop composition (Chassy 2010). These comparative composition studies are used to assess whether levels of nutritionally important components and/or components that can affect the safety of the food and feed have not been altered in a manner that would adversely impact human or animal health.

The compositional studies are conducted using targeted analytical methods that are validated for accuracy and precision and performed under good laboratory practices (GLP) for each specific crop and tissue matrix analyzed. This targeted analysis approach ensures the accuracy and reproducibility of the quantitation of all nutrients and anti-nutrients to provide regulatory agencies with the data required to ensure a safe and nutritious food and feed supply. The data from crop composition studies have repeatedly shown that the GM varieties selected for commercialization are compositionally equivalent to their conventional counterpart (Curran et al. 2015; Herman and Price 2013; Ladics et al. 2015a, b; Parrott et al. 2012; Ricroch 2013; Venkatesh et al. 2014, 2015; Xu et al. 2014). The exceptions are a few cases where the desired trait is an intentional change in composition, such as improved nutrition (Chassy et al. 2008).

Omics (e.g., genomics, transcriptomics, proteomics, metabolomics) refers to profiling technologies that aim to characterize biological systems holistically. Advances in genomics and transcriptomics currently provide high coverage of biological systems, however gene sequence and gene expression data are distal from endpoint phenotypes and, therefore are distant from affecting the levels of nutrients, antinutrients and other factors that contribute to food and feed quality and safety. Proteomics and metabolomics are closer to such endpoint phenotypes, but the diversity of physicochemical properties of the molecules within the proteome and metabolome fundamentally limit the ability of analytical methods to provide full coverage for complex biological systems such as crop plants. Metabolomics combines analytical chemistry with bioinformatics to attempt to comprehensively characterize the small molecules of biological systems. The main metabolomic analytical platforms are mass spectrometry (MS) and nuclear magnetic resonance (NMR). MS is typically more sensitive and provides more coverage of the metabolome than NMR. MS methods are usually combined with a separation technique, such as liquid chromatography (LC), gas chromatography (GC) or capillary electrophoresis (CE), depending on the class of small molecules of interest to be analyzed. However, no single analytical platform and separation technique can comprehensively characterize the full metabolome. Nonetheless, with the continuous development of highly sensitive analytical instruments and improved bioinformatic tools, calls for the use of metabolomics for compositional analysis of GM crops are proposed in order to comparatively assess more metabolites than outlined by the OECD consensus documents (Aguilera et al. 2018; Christ et al. 2018NAS, 2016).

This review provides an overview of: (1) the learnings to date from metabolomics studies of GM and conventional crops; (2) the technical opportunities, challenges and strategies facing implementation of metabolomic analytical tools for GM crop safety assessments; and (3) whether metabolomics can better inform the safety and nutritional assessment of GM crops than the quantitative and validated compositional analytical methods used for more than 20 years as defined by the Codex and OECD guidelines.

### What have we learned from metabolomics studies of GM and conventional crops?

Results from both targeted and untargeted omics technologies have been published that assess the environmental safety and safety of food and feed from GM crops, with reviews summarizing their findings (Ricroch 2013; Ricroch et al. 2011). These reviews summarize 60 omics studies that compared GM to conventional varieties in eight major crops. One key conclusion of these reviews is that conventional breeding and environmental factors have a greater impact on the endpoints measured by the various omics methods (e.g., transcriptomics, proteomics, metabolomics) than from the use of modern genetic modification methods to introduce new traits into crops. A review of the challenges of using omics in food safety assessments of GM crops concluded that untargeted metabolic profiling is unlikely to provide interpretable data that would enhance the already rigorously quantitative comparative assessment of GM crops (Harrigan and Chassy 2012).

As additional studies are published, the results reported in many publications continue to support the conclusion that the effect of trait insertion on the metabolomics of GM crops is small compared to the effect of naturally occurring factors associated with the cultivation, environmental and/or genetic changes arising from conventional breeding practices (Table 1). Environmental factors, such as planting location and season, had a greater impact on the metabolome than genetic background, and the environmental effect was more pronounced with forages than grain (Asiago et al. 2012; Baniasadi et al. 2014; Chang et al. 2012; Chen et al. 2016; Frank et al. 2012; Tang et al. 2017).

**Table 1:** Omics studies published since 2012 that characterize either conventional and/or new GM crop varieties

Citation	Product tested	Analytical and statistical analyses		Field design		Summary
*Metabolomic analysis of conventional crops*						
(Asiago et al. 2012; Baniasadi et al. 2014)	654 grain and 695 forage samples from 50 genetically diverse DuPont Pioneer conventional maize hybrids	GC-TOF–MS, Data analysis: t-Test, PCA, HCA		Six North American field locations, RBD		Environment affected up to 50% of the metabolites compared to less than 2% by the genetic background
(Baniasadi et al., 2014)	50 genetically diverse DuPont Pioneer conventional maize hybrids	LC-LTQ MS, Data analysis: t-Test, PCA, PLS-DA, HCA		Six North American field locations, RBD		Environment had a greater effect than genetic background on a total of 286 (grain) and 857 (forage) metabolites. Additionally, the environmental had a more pronounced effect on forage metabolites than grain metabolites
(Harrigan et al. 2016)	NK603 and corresponding negative segregant varieties	GF-TOF–MS, Data analysis: MB-PCA, ASCA		Three field sites in US, RCBD		Differences between GM and non-GM comparators, even in stringent tests using near isogenic positive and negative segregants, reflected genomic differences associated with conventional backcrossing practices, not the use of biotechnology
(Venkatesh et al. 2016)	50 maize hybrids made by crossing B73 with either nested association mapping (NAM) founder lines or landraces from North and South America	GC-TOF–MS and NMR profiling analysis, Data analysis: PCA, PLS-DA, HCA		Hybrids from controlled pollinations of B73 with NAM lines or landraces were field grown over four seasons and two locations, RCBD		Extensive metabolomic variation in grain when comparing both hybrid sets and also across subpopulations within each hybrid set in patterns consistent with the known genetic and compositional variation of these lines
*Metabolomics analysis of GM crops*						
(Chang et al. 2012)	Insect resistant GM rice (cry1Ac and sck)	RP LC-TOF–MS, Data analysis: PCA, PLS-DA	Three plantings locations in two Chinese provinces		Environmental factors had a greater effect on the rice metabolome than the effect of using genetic modification methods to introduce novel traits	
(Frank et al. 2012)	Insect resistant (Bt) and herbicide tolerant (glyphosate) GM maize	GC–MS, Data analysis: ANOVA and PCA	Two locations in South Africa in 3 years, RBD and two locations in Germany		The majority of metabolomic differences were related to natural variability (e.g., location, season), with few differences being attributed to the use of genetic modification methods	
(Clarke et al. 2013)	Herbicide tolerant (mesotrione) soybean and 49 conventional soybean lines	LC–MS, GC–MS, Data analysis: HCA, PCA	Seeds obtained from USDA and Syngenta. No planting information available		The 169 metabolites profiled showed no significant deviation for the GM line from the natural variations represented by the metabolome of the conventional soybean lines	
(Kusano et al. 2015)	Six commercial conventional soybean lines and three GM (glyphosate-tolerant) lines	GC-TOF–MS, LC-qTOF-MS, CE-MS, ionomics, Data analysis: PCA, OPLS-DA, ANOVA	Two field sites in US, RCBD		Genotypic and environmental (location) factors had the dominant effect on the acquired omics profiles. Conventionally bred higher-yielding soybean genotypes were differentiated from lower-yielding genotypes at both field sites. No distinctions detected between the GM and the conventional lines	
(Chen et al. 2016)	50 genetically diverse DuPont Pioneer conventional maize hybrids	GC-TOF–MS, Data analysis: ANOVA, PCA, PLS-DA, HCA	Six North American field locations, RBD		Environmental factors had a greater impact on the metabolites than genotypic factors	
(Muccilli et al. 2020)	Peel and flesh of a transgenic lemon clone expressing the chit42 gene and exhibiting an increased tolerance to some pathogenic fungi	^1^H, ^13^C and 2D NMR based metabolomics; Data analysis: ANOVA, PCA	Greenhouse experiment		NMR metabolomics identified 34 compounds, among which 20 were common to both tissues. Results showed substantial equivalence of the metabolomics profile of the transgenic clone compared to the wildtype	
(Nam et al. 2016)	cytochrome P450-baseds drought-tolerant GM rice line	Proton-NMR and GC–MS, Data analysis: PCA, ANOVA	Well-watered and drought-stressed conditions grown in greenhouse, RCBD		Results mainly distinguished plants from drought and well-watered plots, rather than ± the GM trait. Drought increased in drought-tolerant rice the levels of gamma-aminobutyric acid (GABA, 244.6%), fructose (155.7%), glucose (211.0%), glycerol (57.2%), glycine (65.8%) and aminoethanol (192.4%)	
(Batista et al. 2017)	Drought tolerant (OsICE1 transcription factor) GM rice variety	Transcriptome and proteomics	Rice seedlings grown under controlled environment. Comparison to negative segregant over eight generations after transformation		The effect of salinity stress on the F6 generation showed that environmental stress caused more proteomic/transcriptomic alterations than trangenesis and transcriptomic and proteomic differences observed in early generations of the GM variety were short-term physiological changes and attenuated through subsequent generations, suggesting that the differences observed in the early generations were related to breeding practices, not the GM trait itself	
(Tang et al. 2017)	50 genetically diverse DuPont Pioneer conventional maize hybrids	LC–MS and GC-TOF–MS Data analysis: PCA, HCA	Eight North American field locations, RBD		The environment affected between 36–84% of the metabolites in forage and 12–90% of the metabolites in grain. By comparison, genotype affected 7% (forage) and 27% (grain) of the metabolites grain, and phenotype affected < 10% (forage) and 11%(grain) of the metabolites	
(Wang et al. 2018)	Insect resistance or glyphosate tolerance GM maize, single and stacked	Transcriptomics, LC-TOF–MS based Metabolomics Data analysis: HCA	Leaf samples from five lab grown plants using seed from various labs		Differences between the GM, single or stacked, and their respective parental lines were fewer than those observed between samples of conventional varieties collected from plants cultivated in different environments (e.g., different Chinese provinces)	

Several recent publications have also demonstrated that experimental design affects the interpretability of metabolomics studies. The results from the studies listed in Table 2 are difficult to interpret due to limitations in one or more study design aspects: (1) not using validated and/or suitably replicated test materials (e.g., samples from single, non-replicated growing conditions), (2) test samples that do not align with the intended topic of investigation (e.g., food/feed safety inquiries that analyze non-consumed plant tissues), and/or (3) lack of data to characterize natural variability of the components that were analyzed.

**Table 2:** Omics studies with study design limitations that affects interpretability of data for safety assessment

Citation	Product tested^2^	Analytical and statistical analyses	Field design	Study limitation	Summary
(Plischke et al. 2012)	“Modena” high amylopectin potato	Proton NMR metabolomics, Data analysis: PCA, PLS-DA	Young (6 weeks old) untreated leaves from several biotic-stress environmental conditions: uninfected, virus infected and during aphid herbivory	Food/feed safety discussion based on omics analysis of non-consumed tissues	GM potato plants showed lower levels of sugars (glucose and sucrose) and phenolic compounds compared to their conventional counterpart, but these differences are compared to those from naturally occurring factors
(Zhou et al. 2012)	Bt (Cry1Ab) GM rice ± insecticide treatment	GC–MS, Data analysis: 2-way ANOVA, PCA, HCA	Over-season leaf samples from a single, non-replicated field trial	Food/ feed safety discussion based on omics analysis of non-consumed tissues Non-replicated field samples	A wide range of metabolites were dynamically varied in both GM and conventional varieties in response to insecticide and most changes correlated with the exposure time, although the lack of adequate replication and controls makes it hard to know if the differences are reproducible and whether differences translate to differences in harvested grain
(Zhao et al. 2013)	Lysine-rich protein (LRP) GM rice with 24% increased lysine	Proteomics	No field data included to determine environment influences	Lack of information on natural variability of measured analytes	Proteomics showed 22 protein differences, derived from 18 unique proteins. Two of the altered proteins were the introduced LRPs, but no protein differences were of concern for safety (e.g., allergens or toxins)
(DiLeo et al. 2014)	17 widely diverse conventional tomato cultivars and three types of RNAi-based delayed ripening GM tomato lines	LC-QTrap-MS Data analysis: PCA, PLS-DA, WGCNA	Greenhouse controlled environment	GM events did not exhibit intended phenotype	Although a small suite of highly correlated compounds accumulated to lower levels in the 21 GM events in the study, these GM events did not exhibit the intended phenotype of delayed ripening, therefore the observed differences cannot be attributed to the introduced trait
(Gayen et al. 2016)	GM blight-resistant rice	2-D proteomics and standard composition analysis	Non-replicated greenhouse productions	Non-replicated samples	No new toxins or allergens and showed a total of 11 proteomic differences (4 up, 7 down)
(Mesnage et al. 2016)	NK603 (glyphosate tolerant) maize	Proteomics and LC–MS based metabolomics Data analysis: PCA, MCIA	No data on the growing season or locations or genetic relatedness of GM and control lines	Lack of information on natural variability of measured analytes Non-replicated field samples	Lack of replication of grain samples, lack of information on the genetic relatedness between the GM line and the non-GM comparator and lack of consideration of natural variability of the components were cited by subsequent publications (EFSA et al., 2017; Eriksson et al., 2018) as deficiencies that question the validity of this study
(Christ et al. 2017)	GM Arabidopsis lines expressing BAR or BAR variants and samples from BAR expressing soybean, canola, mustard and wheat	LC–MS based metabolomics	Young and senescent leaves from growth chamber grown plants	Food/ feed safety discussion based on omics analysis of non-consumed tissues Non-replicated field samples	N-acetylated forms of aminoadipate and tryptophan detected in all BAR-expressing GM plants. Several BAR variants generated with significantly reduced nonspecific activities compared with the wild-type counterpart in GM Arabidopsis plants
(Hao et al. 2017)	Four GM maize lines (overexpressing two different Bt proteins) and two parental lines	LC–MS based metabolomic Data analysis: PCA, PLS-DA	Single fully expanded leaves from seedlings grown in tubs	Food/ feed safety discussion based on omics analysis of non-consumed tissues Non-replicated field samples	56% of the metabolites could be putatively annotated, with purine and glutathione related metabolites showing most of the detected differences, although leaves from other GM events with these Bt genes in other maize germplasm detected a different array of differences – so results do not support conclusions that the differences relate to how Bt genes function
(Hrbek et al. 2017)	MON 89788 soybeans (glyphosate tolerant) compared to a conventional line	LC-qTOF-MS and DART-Orbitrap MS Data analysis: PCA, OPLS-DA	No data on the growing season or locations or genetic relatedness of GM and control lines	Lack of information on natural variability of measured analytes	Differences detected for phosphatidylcholines and minor sugars (e.g., maltol, isomaltol) between GM and conventional soybean varieties, although without data on field replications, genetics, etc., no conclusions possible on whether results are reproducible

Two studies in Table 2 warrant additional discussion of their results. One of these studies reported that two plant endogenous metabolites, aminoadipate and tryptophan, are acetylated in GM plants with the Bialoaphos resistance gene (*bar*), conferring resistant to the broad-spectrum herbicide glufosinate (Christ et al. 2017). However, the results of this metabolomics study are not unexpected since BAR confers glufosinate resistance to plants through its acetylation functionality. The authors reported in the supplemental information that the levels of acetylated tryptophan were around 0.25 and 0.1 nmole/g fresh weight for GM soybean seed and wild type soybean seed respectively. Those values are four orders of magnitude lower than free tryptophan values reported in wildtype soybean seeds (Ishimoto et al. 2010; Kita et al. 2010). Importantly, GM crops with glufosinate resistance have been through extensive regulatory review over decades (ILSI-CERA 2011) and regulatory agencies have consistently concluded that this GM crops are as safe as conventional crops. Therefore, although this metabolomics study detected these two acetylated metabolites in the GM crop seed that was not present in the conventional comparator, the presence of those trace metabolites did not confer anything that was determined to be unsafe to food and feed from these glufosinate-resistant crops.

The second study in Table 2 that warrants further discussion is related to the lack of experimental details in the study prompted publication of secondary assessments of their results and conclusions. In 2016, a multi-omics analysis of the NK603 glyphosate-tolerant GM maize was published in which the authors claimed that NK603 was substantially non-equivalent to its conventional comparator, thereby challenging the many regulatory agency reviews of this GM variety that had concluded that it was “as safe as” conventional maize (Mesnage et al. 2016). Scientists independent of the publication’s authors reviewed the design of this study to better understand the seeming disconnect between its conclusions compared to regulatory agency conclusions (EFSA et al. 2017; Eriksson et al. 2018). These scientists concluded that the conclusions of Mesnage et al. were not supported by their results due to several factors: (1) lack of replication of grain samples that were analyzed, (2) lack of information on the genetic relatedness between the GM line and the non-GM comparator, and (3) lack of data to characterize natural variability of the components that were analyzed. Therefore, any differences that they detected may have been due to environmental or genetic differences.

## What is needed for metabolomics to be ready for risk assessments of GM crops?

As a biomarker discovery tool, metabolomic studies (and other omics studies) and their related statistical analysis strategies are constructed to detect potential differences between a test group and an appropriate comparator to facilitate discovery of metabolic genes and pathways pivotal to key biological processes (e.g., improved crop yield, plant, animal and human diseases) (Ren et al. 2015). Therefore, for discovery purposes, metabolomic studies are a good starting point for additional research to understand whether any detected differences are of sufficient biological significance to warrant further study. By comparison, scientific studies that support regulatory safety assessments and approvals of new products (e.g., GM crops, pharmaceuticals) are the final research studies needed to evaluate the efficacy and safety of the new product before that product can be commercially released for public use.

Regulatory studies, therefore, must reliably characterize biological endpoints critical to ensuring that the product achieves the intended outcomes while assuring that unsafe changes have not been inadvertently introduced (Codex Alimentarius 2009; EFSA 2006). Regulatory agencies have rigorous experimental criteria for data derived from studies used for safety assessment purposes (e.g., US FDA and EPA Good Laboratory Practices, ISO 9000). For regulatory assessments of GM crop safety, a comparative assessment process has been used for more than 25 years (Codex Alimentarius 2009; EFSA 2006). The goal of this comparative assessment is to determine whether components of a GM crop measured by validated analytical methods are statistically different from near isogenic controls that have a history of safe use. One fundamental feature to this comparative assessment is determining whether any detected differences are outside natural variation observed with conventional crops. Also critical is establishing whether any detected change(s) would affect food and feed safety. From 1992 to 2017, 40 countries (including the EU 28, counted as one) have given 1,995 food approvals 1338 feed approvals, and 800 cultivation approvals for 498 events of 29 crops (ISAAA, 2017). It is noteworthy that no approved GM crop has caused any adverse environmental or human/animal health occurrence (ISAAA, 2017; NAS, 2016). Given this robust track record, if metabolomic studies were to be added to GM safety assessments, it would be advisable to use the same experimental criteria in order for them to meet the rigorous end-point requirements for regulatory safety assessment purposes.

### Tools to interpret metabolomics data for GM comparative safety assessments

One key hurdle to using data from omics studies with GM crops, including metabolomics, is the difficulty to assess whether there is any impact on safety in the observed differences amongst the 1000′ s of signals characterized by the untargeted profiling method(s). In 2014, a proposal was published to use a one-class SIMCA (Soft Independent Modelling of Class Analogies) model to quantify omic comparisons (van Dijk et al. 2014). A multivariate one-class classification model was built using transcriptomic profiles of six commercial potato varieties with a history of safe use. The profile of a new test variety would either fall inside the model and be regarded as “similar” to the reference varieties considered as “safe”, or outside the model and be regarded as “different” from the reference varieties, resulting in the need for further safety assessment.

The one-class SIMCA model was recently revised using transcriptomic and metabolomic profiles of a larger set of potato varieties including commercial conventional varieties, experimental varieties and one GM variety (Kok et al. 2019). This study was part of the European GRACE (GMO Risk Assessment and Communication of Evidence) project in which the central objective was to evaluate all aspects of the current EFSA GMO risk assessment procedures. Using the statistical model, the GM variety was found to be “as similar as” the conventional varieties, having no detected differences. The authors submit that this one-class classification model is a more useful tool to assess any potential unintended effects in GM crops compared to the current statistical model assessing both differences and equivalence of GM crops used results from targeted compositional analyses. The authors also cite the metabolomic analysis as being more holistic than the targeted compositional end points recommended by OECD consensus documents for each crop. However, the authors suggest that metabolomic studies with the one-class SIMCA model might be used in a tiered approach where only GM varieties shown to be different from conventional varieties by the current targeted compositional analyses would be subjected to assessment by this one-class omics method.

One proposed advantage to a tiered approach is that it could reduce the need for animal feeding trials with whole foods that are currently an obligatory part of the risk assessment process in Europe, since it is perceived that “omics” analysis provides a more comprehensive assessment of the GM variety for unintended changes than is possible by targeted analyses (Kok et al. 2019). Moreover, it has also been claimed that omics techniques are more sensitive than the 90-day animal feeding studies with whole foods/feeds (Corujo et al. 2019). This claim is from a study using the one-class model with results from transcriptomic, proteomic and metabolomic analysis of grain samples from two insect resistant MON 810 maize varieties compared to their near isolines and compared to several other conventional reference maize varieties. The grain samples used in this study had limited representation of natural variation because they were derived from the GRACE 90-day feeding trials that had involved six conventional maize varieties planted at a single location (Zeljenková et al. 2014). Data were analyzed either by direct comparison of the GM to the conventional materials or by the one-class SIMCA model. Although the threshold for acceptance or rejection for the GM tested variety within the model is unclear, the observed differences between GM and their isolines did not exceed differences typically observed between conventional varieties. It is important to observe, therefore, that the outcome of using the one-model SIMCA analysis of untargeted omics results was no different than the outcome of the targeted compositional assessment of MON 810 grain based on the analytes defined for maize by the OECD that showed that MON 810 was as safe and nutritious as conventional varieties prior to commercialization in the EU and elsewhere (Sanders et al. 1998).

Beyond the observation mentioned above that the one-class model with metabolomics data does not provide insights different from standard statistical analysis of results from targeted compositional studies, two additional scientific questions need to be answered prior to considering implementation of the one-class model metabolomics method. First, can the model identify the metabolomic signals responsible for rejecting a GM variety from the safe classification for further follow-up studies? That would be essential to enable follow-up on whether these noted metabolite differences affect the safety of food/feed from the GM variety. This is critical since most metabolomic studies of crops (Ricroch 2013; Ricroch et al. 2011) and Table 1 have shown that most observed differences are the result of natural environmental factors and yet food and feed have a history of safe consumption, regardless of where it was produced. Second, would a model be constructed for each GM study from commercial varieties grown in the same conditions as the GM and its isogeneic control? Constructing a model for each experiment, “self-contained” model, will allow each new study to take advantage of the continuous development in instrumentation and data processing technologies. However, it will provide only a snap shot of the natural variability captured within each study. If the model is adapted to allow cumulative acquisition of data from “safe” varieties over time, this allows a better representation of natural variability of metabolites captured over multiple years and environments. However, the downside to a cumulative acquisition model is that the methods would need to be locked and would, therefore, lose the ability to integrate new technologies as they are developed.

A similar approach for interpreting metabolomics data for GM comparative risk assessments would be to build crop specific metabolomics databases constructed from conventional varieties with a history of safe use to represent the natural variability of metabolites over different cultivation environments. Differences detected between the GM variety and its near isogenic variety would be evaluated in the context of the natural variability represented in the crop-specific metabolomics database to establish whether any observed differences are outside of the natural variation currently part of the history of safe consumption of foods/feeds from crops. Building such a crop-specific database would require establishing globally accepted standard method(s) and would possibly be needed to be constructed under good laboratory practice (GLP) or ISO protocols for quality assurance. Challenges to construct such a method will be discussed in the following section.

### Challenges with a metabolomics method that captures the diversity of the plant metabolome

A single plant species is estimated to produce tens of thousands of metabolites, far more than those produced by most other organisms (Fang et al. 2019; Kessler and Kalske 2018). Those metabolites are present at a wide range of concentrations and are produced at different developmental stages and in a diversity of tissues or as a response to biotic and abiotic environmental stress. No single extraction method or analytical technique can capture the complete metabolome of a plant because of the physiochemical diversity of their metabolites (e.g., aqueous solubility, degree of ionization, degree of electronegativity, volatility, stability, molecular size/complexity). Multiple extraction systems and analytical platforms are required to approximate full coverage of the metabolome. Attempting 100% coverage would be technically impractical, regardless of the array of methods employed. The analytical platforms used in metabolomics analyses are either based on MS or NMR, with MS being more sensitive than NMR and often coupled with a separation method to increase metabolite coverage and reduce ion suppression.

Sample quenching that stops metabolic activity to preserve the array of metabolites at the time of collection plus the extraction conditions has a great effect on enriching certain classes of metabolites and their stability during extraction (Lu et al. 2017). Lipids and highly hydrophobic compounds are usually extracted in organic solvent, while many other secondary metabolites are extracted in aqueous methanol or aqueous acetonitrile solutions. Primary metabolites are more polar and are usually extracted with aqueous solutions. Other components of plants that are either storage (e.g., starch) or structural (e.g., cellulose, lignin) materials are hard to extract or unextractable, yet they constitute a considerable amount of the overall mass of a plant and require targeted methods for analysis.

Untargeted metabolomics data are highly dependent on the acquisition platform, whether LC–MS, GC–MS or CE-MS as well as the high-resolution mass analyzer used. Each acquisition platform is most suitable for a specific class of metabolites. No single analytical technique will be able to cover the breadth of the plant metabolome. Alternatively, a targeted metabolomics approach would be more applicable when a class of metabolites or a specific pathway of interest is investigated due to the mode of action of the inserted GM gene. Furthermore, targeted metabolomics is a semi-quantitative technique and can be standardized across acquisition platforms using standard substances compared to the relative quantitative untargeted metabolomics.

It is worth noting that the quantitative, validated compositional methods for analytes defined by crop-specific OECD consensus documents already well inform the nutritional quality and safety of food/feed from each crop. Adding more metabolites to comparisons of GM to conventional crops does not necessarily add information that better informs the safety assessment since the OECD documents are based on close examination of the components of each crop that contribute to food and feed safety and nutrition, often derived from millennia of human experience with the crop. In addition, many of the important end points identified by the OECD are not captured by metabolomic profiling, such as proximate, minerals and fiber analysis.

### Standardization of the metabolomics method

The metabolomics standards initiative (MSI) was introduced in 2007 to align on the minimal reporting standards for a metabolomics study including the plant biology context and parameters for chemical analysis (Fiehn et al., 2007a, b; Sumner et al. 2007). For example, a minimum set of reporting standards for metabolite identification have been proposed (Sumner et al. 2007) and include four categories: (A) Confident identifications are based upon a minimum of two different pieces of confirmatory data relative to an authentic standard. (B) Putatively annotated compounds based upon physicochemical properties and/or spectral similarity with public/commercial spectral libraries. (C) Putatively characterized compound classes based upon characteristic physicochemical properties of a chemical class of compounds, or by spectral similarity to known compounds of a chemical class. (D) Unknown compounds.

Driven by the progress in clinical metabolomics, quality control and quality assurance measures proposed for untargeted metabolomics studies have been recently discussed (Beger et al. 2019; Broadhurst et al. 2018; Dudzik et al. 2018). QC samples and QC blanks can be used for system suitability to monitor the performance of the metabolomics workflow and ensure that high quality data is collected. Use of multiple internal standards and pooled QC samples can help compensate for intra-study bias and drifts in instrument response. An international interlaboratory ring trial for targeted metabolomics and lipidomics heighted the importance of using system suitability and quality controls to understand and control variability of targeted mass spectrometry-based metabolomics (Thompson et al. 2019).

Development of a global standard for reference materials for inter-study and inter-laboratories comparison is required to compare data sets between experiments. Furthermore, these global standards will help to address the need to understand natural variability of metabolites assessed by untargeted metabolomic studies. It is noteworthy that the majority of signals in a typical MS-based metabolomics study are for unknown metabolites. Therefore, having standardized methods will help to determine the range of variability for these unknowns to identify when a specific new variety, such as a new GM crop, might have levels of the metabolite that are outside of the natural range. Even with such information, however, it will be difficult to assess the food/feed safety risk that might be associated with unknown metabolites unless efforts and resources are allocated to identify the unknown. Furthermore, since data generated using standardized methods needs to be reproducible over time and laboratories, the studies might be best if conducted under GLP or ISO standards. Therefore, initiatives are needed to align global standardized workflows for sample processing, data acquisition and data processing for the many available hyphenated analytical platforms manufactured by multiple vendors.

Structural elucidation of small molecules continues to be one of the major challenges in untargeted mass-based metabolomics studies. Although significant progress had been made in instrumentation development and software computation capabilities (Barupal et al. 2018; Dias et al. 2016; Viant et al. 2017), this challenge still limits the interpretation of metabolomics data to understand complex biological systems, to map metabolites to specific pathways, and to predict metabolic perturbation. An active area of research is the development of spectral databases and software tools to annotate unknown compounds by searching available tandem MS databases (Blazenovic et al. 2018; Vaniya and Fiehn 2015). Several metabolomics spectral databases are available to assist in metabolite identifications. Examples are the Human Metabolome Database (Wishart et al. 2018), The Metabolomics Workbench (Sud et al. 2016), GOLM Metabolome database (Fernie et al. 2004), the Plant Natural Product Library (Lei et al. 2015), the FiehnLib (Kind et al. 2009) and the KNApSAck Family Database (Afendi et al. 2012). The confidence in MS/MS-based annotation is impacted by the parameters of instrumental data acquisitions, data processing and library scoring algorithms (Kind et al. 2018). Another approach is to annotate unknown compounds by in silico fragmentation of existing structure databases using quantum chemistry and machine learning methods.

## Conclusion

The process of developing a GM crop includes extensive molecular characterization to ensure that only a single copy of an intact DNA is inserted in the genome and without disruption of endogenous genes. After a GM event containing the desired DNA insert is chosen, the DNA insert is introduced into well-characterized, conventionally bred elite varieties through multiple backcrossing steps. The resulting offspring will theoretically contain > 99% of the DNA from the elite parent and < 1% from the GM event, which will then undergo extensive phenotypic characterizations and safety assessments prior to commercialization as required by global regulatory authorities (Glenn et al. 2017). Therefore, it is highly unlikely that, even if an unintended change has occurred from the plant transformation process, this unintended DNA change is present in the genome of a commercialized GM crop. Nevertheless, Codex and regulatory requirements are in place largely to assess the safety of food and feed from GM crops with both the intended change and the possible presence of such unintended changes.

The Codex Guidelines to assess the safety and nutritional quality of foods and feeds from GM crops is because no food or feed has absolute safety. Therefore, a comparative process is followed in which the composition of the GM crop is compared to a near isogenic conventional counterpart (Codex Alimentarius 2009; Paoletti et al. 2008; Prado et al. 2014; Privalle et al. 2012). Over 25 years, no examples of unsafe unintended alterations have been documented for GM crops. For example, livestock animals consume 70 to 90% of GM crop biomass globally, and in the USA, billions of animals have been eating GM feed for over two decades (Van Eenennaam and Young 2014). As reviewed, animal productivity data over the time in which GM crops have been adopted in the USA as the predominant source of feedstuffs for commercial livestock populations has not revealed any unfavorable or perturbed trends in livestock productivity. This review notes, for example, that more than 95 billion broiler chickens were raised in the US from 2000–2011, with nearly 100% of their diet (excludes vitamin and mineral supplements) coming from feed from GM crops (maize and soybean), and yet no adverse consequences have been documented by US regulatory agencies that oversee animal agriculture. The vast level of real-world experience with consumption of foods from GM crops is consistent with the lack of any observed effects in laboratory-scale animal feeding studies, including sub-chronic and chronic feeding studies funded by the European Commission such as GRACE (GMO Risk Assessment and Communication of Evidence) (Zeljenková et al. 2016, 2014) and G-TwYST (GM Plant 2 Year Safety Testing) (Steinberg et al. 2019). Numerous long-term and multi-generation feeding studies with various livestock animals have consistently demonstrated healthy animal growth and reproduction, results that confirms Codex-defined assessment of the safety of foods/feeds from GM crops. Many of those feeding studies are reviewed by (Flachowsky and Reuter 2017; Snell et al. 2012).

In the current state, there are major hurdles ahead of utilizing untargeted metabolomics in regulatory safety assessments and thus are not considered useful in the risk assessment of GM crops or gene-edited crops (Delaney et al. 2019; Fedorova and Herman 2020). For hypothesis driven risk assessment, quantitative crop-specific targeted compositional analysis (Codex Alimentarius 2009; EFSA 2006) is central to safety reviews of food and feed from GM crops. For GM crops with traits known to modify metabolic pathways, targeted quantitative metabolomics could provide additional useful information for safety and nutritional assessment (Chassy et al. 2008). However untargeted metabolomics, by definition, are not hypothesis driven and are best suited for discovery research purposes. Moreover, the perception that untargeted metabolomic methods provide comprehensive analysis of all classes of metabolites is not supported by the current state-of-the-art. As reviewed, most untargeted metabolomics studies use multiple analytical methods to assess different classes of metabolites. Furthermore, assessing the differences found by untargeted metabolomics studies comparing GM crops to their near isogenic conventional counterpart varieties has several technical hurdles to overcome. First, since metabolomic studies have shown that environment and conventional plant genetics significantly affect the metabolome of plants, it is critical to adequately characterize the natural variability of metabolites prior to being able to interpret any observed differences between a GM variety and its conventional counterpart. Second, tools, such as the “one class” statistical model discussed above to analyze the copious amounts of metabolomics data from such studies, have not been fully developed or tested to implement in food and feed safety studies. Third, since currently there is no body of knowledge that represents the natural variability of crop metabolomics, generating such data will require the development of standardized methods. The efforts by the clinical metabolomics community have shown that developing standardized methods is currently still far from reach. Finally, structural identification of metabolites by metabolomic studies remains one of the major challenges in the field. Given most published metabolomic studies to date are doing well to have at most 50% of the acquired signals being putatively identified, this indicates that metabolomics techniques are not yet appropriate for utilization for GM crop food and feed safety studies since it would be impossible to know if any of the observed differences for unknown metabolites have any relationship to the nutritional or safety of food/feed from the GM crop.

This weight of evidence indicates that unintended effects of GM crops on feed and food has not been shown to be a concern. Nevertheless, calls to use metabolomics for supplementing compositional analyses of GM crops to assess “potential unintended effects” are promoted though unfounded concern that genetic modification will somehow give rise to unsafe food or feed products.
